# A portable optical reader and wall projector towards enumeration of bio-conjugated beads or cells

**DOI:** 10.1371/journal.pone.0189923

**Published:** 2017-12-21

**Authors:** Macdara T. Glynn, David J. Kinahan, Niamh A. McArdle, Jane L. Kendlin, Triona M. O’Connell, Jens Ducrée

**Affiliations:** 1 FPC@DCU, Fraunhofer Project Centre at Dublin City University, Dublin City University, Glasnevin, Dublin 9, Ireland; 2 School of Physical Sciences, Dublin City University, Glasnevin, Dublin 9, Ireland; 3 School of Biotechnology, Dublin City University, Glasnevin, Dublin 9, Ireland; Texas A&M University College Station, UNITED STATES

## Abstract

Measurement of the height of a packed column of cells or beads, which can be direclty related to the number of cells or beads present in a chamber, is an important step in a number of diagnostic assays. For example, haematocrit measurements may rapidly identify anemia or polycthemia. Recently, user-friendly and cost-efficient Lab-on-a-Chip devices have been developed towards isolating and counting cell sub-populations for diagnostic purposes. In this work, we present a low-cost optical module for estimating the filling level of packed magnetic beads within a Lab-on-a-Chip device. The module is compatible with a previously introduced, disposable microfluidic chip for rapid determination of CD4+ cell counts. The device is a simple optical microscope module is manufactured by 3D printing. An objective lens directly interrogates the height of packed beads which are efficiently isolated on the finger-actuated chip. Optionally, an inexpensive, battery-powered Light Emitting Diode may project a shadow of the microfluidic chip at approximately 50-fold magnification onto a nearby surface. The reader is calibrated with the filling levels of known concentrations of paramagnetic beads within the finger actuated chip. Results in direct and projector mode are compared to measurements from a conventional, inverted white-light microscope. All three read-out methods indicate a maximum variation of 6.5% between methods.

## Introduction

Enumeration of cells, and particularly of specific cell sub-populations, constitutes an important step in diagnostic assays and also in life science research. Cell-counting technologies, based on flow cytometry, are well established and have been widely used in research and clinical laboratories for decades [1]. However, these cytometers remain expensive and complex instruments suitable only for use in well-supported centralised laboratory environments. In order to make cell-enumeration based assays available at the ‘point-of-use’, in particular in low-resource settings, rugged, robust and low-cost systems for integrated cell-sorting and analysis are needed. While lacking the general flexibility of conventional flow cytometers, a range of microfluidic systems already address specific applications ranging from blood haematocrit measurement [2] to differential blood counts [3]. Within the microfluidics community, two cell sorting / enumeration applications have been of particular interest; isolation and identification of Circulation Tumour Cells (CTCs) [4, 5] for early-stage cancer diagnosis, and enumeration of CD4+ cells for HIV diagnosis [6].

Metastatic cancer is recognised as the most serious stage of the disease and is responsible for approximately 90% of related deaths. Via the vascular or lymph systems, CTCs reach distal sites where they may seed metastasis [7] [8]. Thus, detection of CTCs in a ‘liquid biopsy’ has great potential for supporting disease prognosis and therapy monitoring. Methods of CTC analysis include nucleic-acid testing as well as investigation of cell morphology and surface biomarkers [9, 10]. In a typical first step, the sub-population of rare CTCs must be isolated from whole blood based on size-filtration or biological markers. Separation of target cells with specifically biofunctionalized paramagnetic beads has already shown particular promise [11]. Kirby *et al*. [12] recently utilised a centrifugo-magnetophoretic isolation technique to enumerate cells from whole blood spiked with EpCAM positive cells. Of particular relevance to this work, they described the relationship between the height of the packed cell conjugate to the initial concentration of EpCAM positive cells loaded onto the chip.

Since its emergence in the early 1980s [13, 14], the prevalence of HIV (human immunodeficiency virus) infection and the associated pathology of AIDS (acquired immunodeficiency syndrome) in countries with poor health-care infrastructures has steadily increased to pandemic levels. It is estimated that HIV / AIDS has led to the death of 36 million people[15] and that, in 2016, 36.7 million [30.8 million – 42.9 million] people globally were living with HIV [16, 17]. It is characterised by a strong geographic focus; 2012 statistics reflect that Sub-Saharan Africa accounts for 71% of those living with HIV and 75% of the AIDS-related deaths [15]. Globally, the number of new HIV infections, and also the number of HIV related deaths, has decreased steadily since 2016 and 2005, respectively; thus, the overall increase in the population living with HIV is reflective of extended life expectancy rather than a worsening of the pandemic. A considerable part of this encouraging trend is attributed to improved access to anti-retroviral therapies (ART) [18]. By 2016, 19.5 million people were accessing antiretroviral therapy [16] with five of seven of them living in sub-Saharan Africa [19]. It is therefore clear that the HIV Treatment Cascade (sometimes called the HIV / AIDS Care Continuum) [20, 21], of which the first step is diagnosis, should re-focus on the resource-limited settings where the disease is most prevalent.

Diagnosis is the first stage of the treatment cascade, and is sometimes referred to as ‘aware of status’. This is followed by 1. link to medical care, 2. continuation of medical care, 3. ART, and 4. viral suppression. Yet, this first diagnostic step remains a major bottleneck for initiation of treatment in Sub-Saharan Africa; in 2012 only 51% of those living with HIV were aware of their status while, strikingly, in certain countries it is even as low as 10% [15]. A primary reason for this shortfall is the failure of existing solutions to meet the WHO ASSURED [6] criteria: Affordable, Sensitive, Specific, User-friendly, Rapid / Robust, Equipment-free and Deliverable [6]. Often, this challenge has been addressed by using technologies originally designed for use in laboratory environments, with limited success so far. The shortcomings of this approach, slow time to result owing to the poor communication infrastructure in Sub-Saharan Africa, has resulted in high patient withdrawal from the treatment cascade – the so-called ‘loss to follow up’ [22, 23].

The recent development of targeted ‘Point-of-Care’ (PoC) instrumentation, which is specifically designed to meet the ASSURED criteria for deployment in remote settings with poor health infrastructure, shows great potential to remove the ‘aware of status’ bottleneck [24]. Jani *et al*. report that ‘loss to follow up’ decreased from 64% to 33% through the introduction of PoC testing at a clinic in Mozambique [25]. Much effort has been made to develop microfluidic solutions to address this point-of-care need [26–28]. Glynn *et al*. reviewed prototype and pre-commercial PoC platforms which have been designed for decentralised HIV diagnostics [6].

The concentration of CD4-expressing T-helper cells (T_h_-cells) in the blood of a patient is considered a reliable measure of the status of HIV infection. While the number of CD4+ cells varies according to demographic, environmental and health factors [29, 30], an uninfected and healthy person will generally have more than 1200 T-cells μl^−1^ of whole blood. As CD4+ cells are targeted by the HIV virus, the presence and stage of infection can be indicated by the reduction in these cells. The previous threshold recommended for initiating ART at 500 cells μl^−1^ [15] was recently updated to provide strong recommendation for initiation of ART for all adults living with HIV, no matter what their CD4+ cell count [31]. Similar, but conditional recommendations were made for children older than one year and adolescents. The guidelines also recommend, where necessary, prioritising those with a CD4+ count below 350 cells μl^−1^.

Recently, Glynn *et al*. [29] presented a magnetophoretic chip optimised for usage in resource poor-settings. Unlike other magnetophoretic separation techniques which often require secondary forces [32, 33] or complex support equipment [11, 34], the chip is solely actuated by (manual) finger pressing. Furthermore, the uniform depth of its microfluidic features lends itself to cost-efficient mass replication by casting, embossing or injection moulding. Additionally, the platform does not require complex manufacturing steps associated with integration of functional material such as dissolvable barriers [35]. This chip functions by deflecting paramagnetic beads, functionalised with anti-CD4+ antibodies, into a dead-end funnel. These beads, both free and bound to CD4+ cells, aggregate to a filling height serving as a good estimate of the number of CD4+ cells present in the patient sample which may be read out directly by engraved hatch marks.

**Fig 1:**
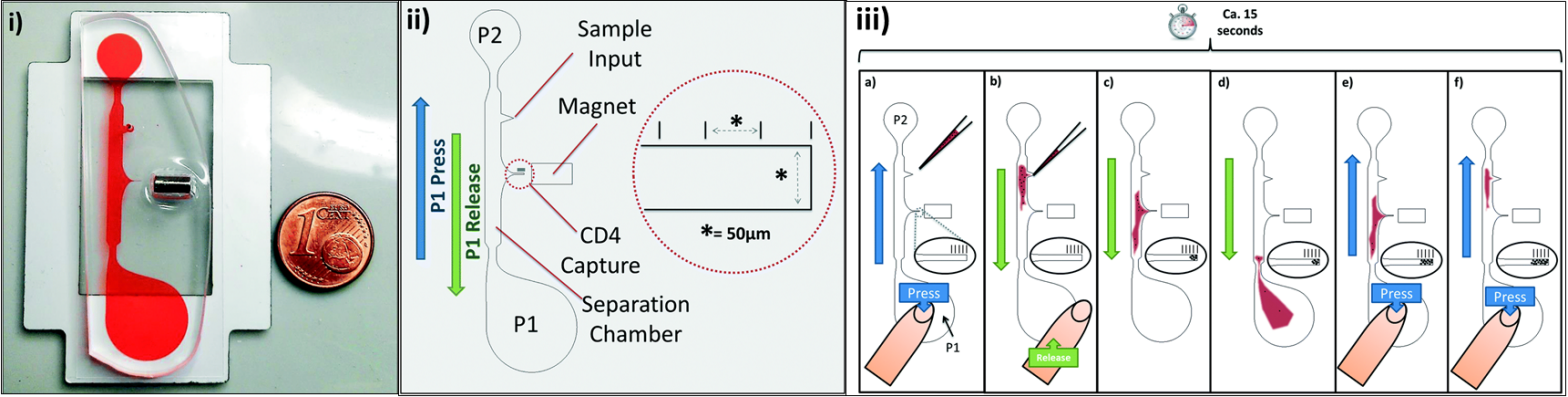
The finger-actuated CD4 enumeration chip previously introduced by Glynn *et al*. [29]. (i) The chip filled with dyed water to highlight its geometry. (ii) Schematic of chip and (iii) chip operation. To operate, the chip is first primed with buffer through degas driven flow. To load, the P1 chamber is depressed and sample is pipetted into sample input port. The P1 chamber is then released and, as the chamber relaxes to its earlier shape, the sample id drawn through the chip and past the capture chamber. Repeated pressing and release of the chamber reciprocally pumps the sample through the separation chamber. Figure is reproduced from Glynn *et al*. [29] with permission of The Royal Society of Chemistry (RSC).

In this paper we present an optical reader which is compatible with the chip introduced by Glynn *et al*. [29] (Fig 1). This modular device is composed of four parts. Its chassis holds an objective lens for magnifying the bead / cell conjugate, and a diffusion plate provides sufficient contrast when using ambient light to view the test result. The chip is mounted on a moveable holder which can be moved along the objective lens optical path by adjusting a screw adapted from a commodity bolt. The lens holder of the objective can be removed to facilitate exchange of alterative lenses. The operating mechanism can be altered by substituting the diffusion plate with a powerful and inexpensive white Light Emitting Diode (LED). This light source projects the shadow of cell / bead /particle conjugate along an ancillary scale on the chip, onto the wall of a (moderately) darkened room.

In this study our reader measures height of bead conjugate in both ‘direct interrogation’ and ‘projector’ modes, and, for comparison, *via* a standard inverted microscope. The independently obtained measurements show good agreement and thus demonstrates potential for future application to HIV diagnostics or CTC detection.

## Experimental methods

### Chip manufacture

The microfluidic chips are prototyped using the method previously described by Glynn *et al*.[29]. First, a master is created from SU-8 using photolithography [33, 36]. The chip is formed from polydimethylsiloxane (PDMS) (Dow Corning, MI, USA) mixed at a ratio of 10:1 between base and curing agent. To secure the magnet, a cavity is created in the mould using a 3D printed jig [33]. Two moulds were used, each of which produced four chips. Chips were randomised before testing.

When cured, the PDMS is cut from the mould and a sample loading hole is defined using a dot punch. Following this, the PDMS slab is placed on a plasma-treated standard glass slide and bonded by stiction. The PDMS-glass hybrid was then secured, using pressure sensitive adhesive (PSA – Adhesives Research, Ireland), to a supporting bracket cut from polymethylmethacrylate (PMMA) (Radionics, Ireland) by a CO_2_ laser writer (Epilog Zing, USA).

Prior to use, the chips are kept under vacuum for at least 1 hour. To prime, a large drop of priming buffer composed of phosphate buffered saline (PBS) pH 7.4, 0.1% w/v bovine serum albumin (BSA) and 1 mM ethylenediaminetetraacetic acid (EDTA) covering the inlet for sample-loading was deposited on the surface of the PDMS. This buffer is drawn into the channels by degas flow [37]. Cylindrical magnets (NdFeB N45, Supermagnete, Germany) with a diameter of 3 mm and a height of 6 mm were placed in the moulded cavities prior to loading the sample on chip.

### Reader manufacture and use

**Fig 2:**
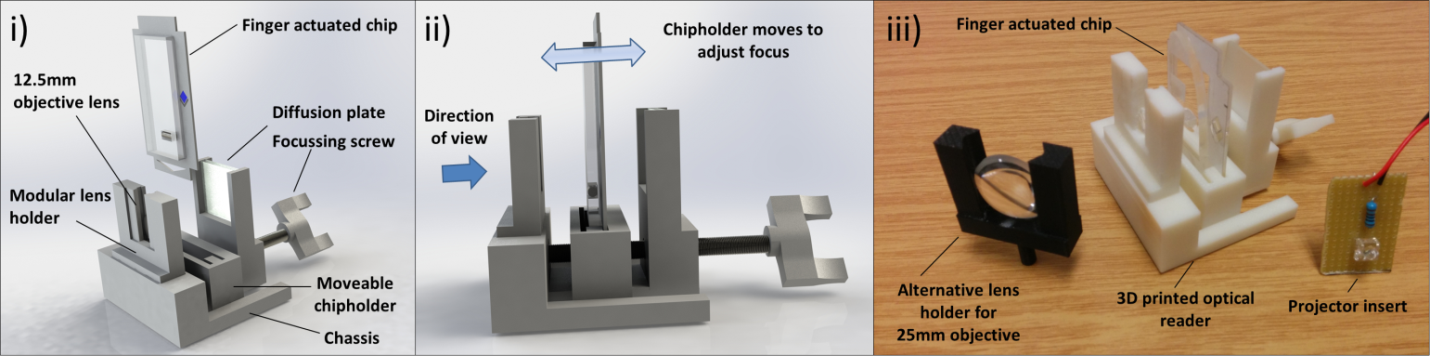
The optical reader for CD4 cell enumeration. (i) The low-cost device is 3D-printed from four separate parts. Additional parts are a threaded screw, objective lens and diffusion plate. The finger actuated chip is inserted into a moveable chip holder. Its chip location relative to the objective lens, and thus the focus, can be finely adjusted by turning the focusing screw. (ii) The chip is interrogated by looking through the objective. The packed height of CD4+ cells and bead conjugate can be estimated from graduated hatch marks. Alternatively, the hatching can be calibrated as ‘treat’ or ‘no treat’ based on clinical guidelines. (iii) Image of the optical reader. Also in the image is an alternative lens holder for larger objectives. Additionally, a ‘Projector insert’, a powerful, low cost LED powered by a 3V battery, can be placed into the reader in place of the diffusion plate. In this case, the shadow of the packed cells and graduated hatch marks can be projected against a wall or floor.

As described above, the reader (Fig 2) is composed of a modular chassis with a moveable chip holder. The parts were first modelled using computer aided design (CAD) software (SolidWorks 2013, Dassault Systèmes, France). They were created using a Dimension μPrint Plus (StrataSys, USA) fused deposition modelling (FDM) 3D printer. The reader is designed so that, when loaded with a chip, the CD4+ capture channel is aligned with the light path between the objective lens and the diffusion plate. To provide an accurate focus, a low-cost masonry bolt (M10) translates the chip-holder by 1.5 mm per full rotation, thus enabling sensitive focussing. Two different, plano-convex lenses were used (both from Edmund Optics, York, UK); one with a 12.5 mm diameter and the other with a 25-mm diameter. Both lenses had a focal length of 30 mm and were found to have equal performance.

**Fig 3:**
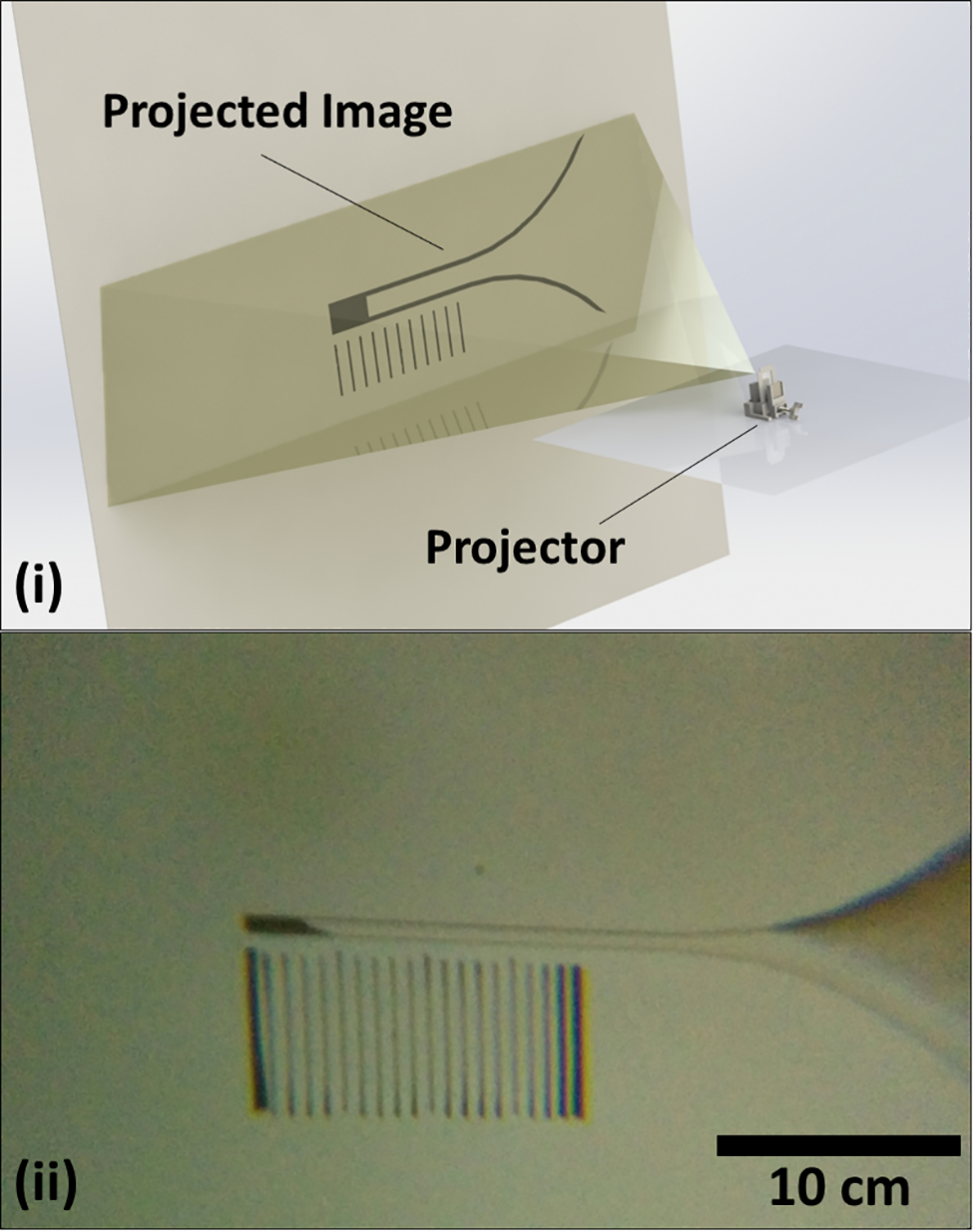
Projector mode operation. (i) Schematic of the reader in projector mode. A shadow of the read chamber can be projected onto a wall from a distance of ~1 m and can easily be discerned in a dim or dark room (ii) an image (acquired using a smartphone) of the read chamber shadow projected onto a wall. This is read as ‘5’ relative to the graduated markings. The projected image is approximately 50 times larger than the read chamber.

Two identical chip-readers were manufactured for this study. One is configured for direct interrogation; for the second device operating in projector mode (Fig 3), the diffusion grating is replaced by a high-power white LED (Radionics, Ireland). Although a single reader can easily be converted from one mode to the next, using two readers for the current experiments allows comparison of read modes without the need for repeated reconfiguration which might introduce systematic errors. The 12.5-mm and 25-mm diameter lenses were used for projector mode and direct testing, respectively.

### Sample preparation and experimental methods

The reader was characterised by testing the system with non-fluorescent magnetic beads (CD14+ tagged beads, Dynabeads^®^ T4 Quant Kit, Thermo-Fischer Scientific). The sample preparation methods used were previously described by Glynn *et al*. [29].

Bead packing is first measured using the ‘direct interrogation’ optical reader by the user conducting the experiments. The chip is then immediately transferred to the reader operating with shadow projection on the wall. In both cases, the height is estimated by the user by rounding the highest visible peak up to the next notch. The chips are then imaged using an Olympus IX81 inverted microscope (Olympus, Tokyo, Japan) by another experimenter. The user again measures the packed bead height using the scale bars integrated into the chip. A consistent rule is applied where the reading is rounded up from the highest point.

## Results

**Fig 4:**
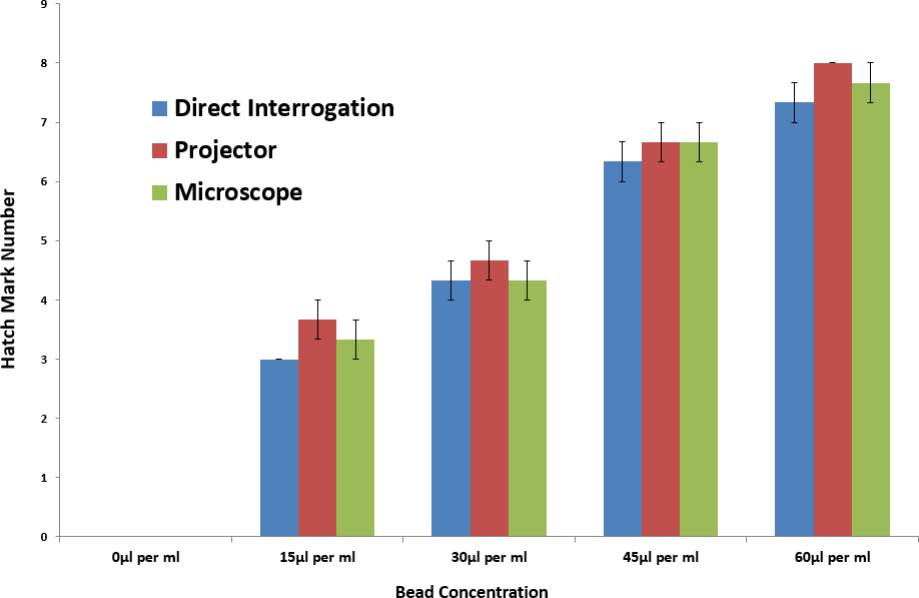
Results acquired using the hand-held reader (n = 3).

As can be seen in Fig 4, the three interrogation methods show good agreement with each other. Results were acquired for different bead concentrations using the three different interrogation methods with a maximum variation within a single bead concentration of 6.5% (where the range is 0-8 hatches). It can also be noted that the packed bead height has an approximate linear relationship with bead concentration for all readings except where no beads are present. We believe this deviation is caused by non-planar agglomeration of the beads in the read chamber. For example, the image in Fig 3(ii) is read as ‘5’ where the packed beads only fill the entire chamber beside the ‘3’ mark. This net effect is to consistently (by ~1.5 marks) over-estimate the height of the packed bead conjugate; except, of course, where no beads are present.

## Conclusions

We have presented an optical device that can be used for interrogating a previously published finger-actuated magnetophoretic chip. The reader works consistently in two different modes. In the first mode, bead counts can be obtained by direct ‘eye-balling’; in the other mode, a shadow of the measurement chamber is projected on to a nearby surface. Either method shows good agreement with measurements made by (blinded) experiments using a commercial microscope. In particular, in a darker environment, the projector proved to be user-friendly and the images are easy to interpret.

The system prototype costs approximately €60 in materials (approx. €30 for the optical lens, €5 for masonry bolt and projector electronics and approx. €25 for 3D printing material and machine time). However, towards mass production, it can be envisaged that the plastic components, and also the lenses, can be manufactured by cost-efficient injection moulding. Indeed, there is potential these components could be moulded as one contiguous part. Thus, we estimate that the cost of this fully re-usable instrument could be as low as €10.

As a next step, we intend to pair this reader with a low-cost smartphone [38] running a custom image analysis software to permit quantitation of the packed bead height in the finger actuated chip. In addition, there is potential to performance-optimise the finger-actuated chip with the focus of optimising system (i.e. chip and reader) performance. For example, by reducing the depth of our capture chamber, the filling ‘height’ of the packed beads will show a greater change with concentration of beads / cells / particles. Combined with a next-generation finger actuated chip, optimised for ease-of-use by automating all steps of sample preparation, this robust and cheap modular technology has the potential to form part of the HIV treatment cascade in resource-poor settings. Similarly, with minor modification, the approach could also be adapted to function with other CTC detection platforms such as the one previously described by Kirby *et al*. [12].

## Supporting information

S1 File ESI_Data. This file contains the data acquired during experimentation.
(XLSX)
